# Wernicke Encephalopathy Presenting with Dysphagia: A Case Report and Systematic Literature Review

**DOI:** 10.3390/nu14245294

**Published:** 2022-12-13

**Authors:** Amalia Cornea, Irina Lata, Mihaela Simu, Elena Cecilia Rosca

**Affiliations:** 1 Department of Neurology, Victor Babes University of Medicine and Pharmacy Timisoara, Eftimie Murgu Sq. No. 2, 300041 Timișoara, Romania; 2 Department of Neurology, Clinical Emergency County Hospital Timisoara, Bd. Losif Bulbuca No. 10, 300736 Timisoara, Romania

**Keywords:** wernicke encephalopathy, thiamine, dysphagia

## Abstract

Wernicke encephalopathy (WE) is a well-known neurological condition caused by thiamine (vitamin B1) deficiency that occurs in both alcoholic and non-alcoholic populations. We aimed to report a case of a patient with WE who presented with dysphagia and dysphonia and later developed typical symptoms of thiamine deficiency and to conduct a systematic review of the literature on this rare presentation of WE. We searched two databases (PubMed and Scopus) and included publications up to November 2022. We found 12 cases of WE and dysphagia, aged between 12 and 81 years; swallowing problems presented at the onset in nine patients (including the current case report). Our findings suggest that thiamine deficiency should be suspected in patients with dysphagia of unknown cause, even in the absence of alcohol abuse. In contrast to most WE patients, the majority of patients included in this review presented with dysphagia at the onset of their disease, even in the absence of the classic triad of cognitive impairment, ataxia, and oculomotor abnormalities, indicating that there could be varying susceptibilities to clinical manifestations of thiamine deficiency in different brain regions.

## 1. Introduction

Wernicke encephalopathy (WE) is a well-known neurological condition caused by thiamine (vitamin B1) deficiency that occurs in both alcoholic and non-alcoholic populations [1,2]. Although encephalopathy is often a consequence of alcohol abuse, it may also occur in patients with any disease that causes malnutrition. In non-alcoholic patients, it has been reported to be associated with various pathologies, including digestive neoplasms [3,4,5] and other gastrointestinal diseases [6,7], hyperemesis gravidarum [8,9], prolonged parenteral feeding [10], and fasting [11,12]. 

The classic WE symptoms consist of a triad of confusion, ocular abnormalities, and ataxia [2]. However, these clinical signs may not all be present in up to 90% of patients [13]. 

Characteristically, the pathological lesions of WE are distributed at the levels of the mammillary bodies, medial thalamic nuclei, hypothalamus, superior cerebellar vermis, periaqueductal gray matter, and midbrain tegmentum. The distribution of lesions explains the symptoms of WE, which, in addition to the triad of confusion, ocular abnormalities, and ataxia, can include vestibular paresis, autonomic dysfunction (including postural hypotension and hypothermia) [1,14], and urinary dysfunction [15]. In some cases, depending on the degree and duration of thiamine deficiency, WE may be accompanied by dry beriberi symptoms, including peripheral neuropathy with paresthesia, patchy sensory loss, weakness, and gait abnormalities [16]. Patients may also present with areflexia, foot drop, and wrist drop [17]. 

Thiamine is primarily stored in the liver; however, the body stores minimal quantities of vitamin B1, and in the case of a deficient diet, severe depletion occurs within approximately 18 days [18]. The serious consequences of thiamine deficiency warrant a high index of suspicion, especially in patients at risk, and a low threshold for treatment. If thiamine is administered parenterally early in the disease course, the patient’s symptoms improve; however, if therapy is delayed, thiamine deficiency can be fatal. 

We aim to present a case of a patient with WE who presented with dysphagia and dysphonia and later developed the classic symptoms of thiamine deficiency. We hypothesized that dysphagia could be a presenting symptom of WE. Therefore, we performed a systematic literature review, aiming to expand our knowledge of the clinical spectrum of the disease and its diagnostic approach to ultimately improve the quality of medical care for patients with WE.

## 2. Case Report

A 64-year-old woman was admitted to hospital with a one-week history of dysphagia, followed by dysphonia, diplopia, and ataxia that developed four days prior to admission and showed progressive deterioration. Her medical history consisted of hypertension and depressive disorder, with episodes of anorexia. In addition, three weeks before admission, she had an acute respiratory illness with fever, rhinorrhea, and dry cough. The patient denied any alcohol or illicit drug use. 

On admission, the neurological signs consisted of severe gait and limb ataxia, absence of deep tendon reflexes, bilateral plantar indifference, paresthesia in the upper and lower limbs, impaired vibration sense, bilateral lateral rectus palsy, horizontal nystagmus, dysphagia, and dysphonia. 

Physical examination revealed a normal heart rate of 78 beats/min and mild hypertension of 152/80 mmHg at admission. Laboratory tests revealed mild leukocytosis (10,800/µL; normal range 4000–9500/µL) and elevated erythrocyte sedimentation rate (45 mm/1 h; normal range 2–20 mm/1 h), serum C-reactive protein (28 mg/L; normal range 0–10 mg/L), and fibrinogen (478 mg/dL; normal range 200–393 mg/dL). Tests also revealed elevated plasma aspartate aminotransferase (ASAT) (40 U/L; normal level < 36 U/L) and slightly elevated serum potassium (5.4 mmol/L; normal < 5.1 mmol/L). Plasma proteins were normal, and serum albumin was at the lower limit of the normal range (3.0 g/dL; normal range 3.0–5.0 g/dL). In addition, serum uric acid was slightly below normal (2.2 mg/dL; normal range 2.6–6.0 mg/dL). Thyroid function was mildly impaired (with an FT3 fraction of 0.165; normal range 0.55–4.78 mU/L), but TSH and FT4 were within normal limits. A detailed list of the paraclinical investigations is presented in the Supplementary Material (Table S1).

Brain computed tomography (CT) showed only mild cortical atrophy. Brain magnetic resonance imaging (MRI) revealed a few lacunar infarcts in the frontal white matter, mild cortical atrophy, and left otomastoiditis. 

Because there were brainstem signs, ataxia, and sensory symptoms, along with diminished tendon reflexes, Miller–Fisher syndrome was suspected. However, anti-ganglioside antibodies, including GQ1b, were negative. Nerve conduction studies revealed mild sensory and motor polyneuropathy and excluded polyradiculoneuropathy. 

The patient received intravenous thiamine (200 mg/day for 5 days, followed by 100 mg/day for 10 days), intravenous ceftriaxone (2 g/day for 10 days), aspirin, statins, and anti-depressive medication. Three days after thiamine initiation, the dysphonia, dysphagia, and right abducens nerve palsy had resolved, along with left abducens nerve palsy improvement. Furthermore, her ataxia and paresthesia had improved considerably. Neuropsychological examination revealed mild memory and concentration problems. 

Fifteen days after admission, the patient showed only mild left sixth cranial nerve palsy and mild ataxia, and she was discharged. The tingling sensations in her upper and lower limbs had subsided, and her tendon reflexes were normal.

## 3. Literature Review

### 3.1. Materials and Methods

This systematic review was performed following the guidelines of the Preferred Reporting Items for Systematic Reviews and Meta-Analyses (PRISMA) for scoping reviews [19,20,21,22] and the current recommendations on the synthesis of case series and case reports [23].

The research question was defined based on the Population, Concept, and Context (PCC) of the review, as recommended by the Joanna Briggs Institute [20]:

- Is dysphagia a symptom of WE?
- If yes, what is the timing of the onset of dysphagia? Could it be the presenting symptom of WE?

We performed a computerized bibliographic search from inception to November 2022 on MEDLINE/PubMed and Scopus. Furthermore, we checked the reference lists of relevant research papers in order to identify any possible additional studies. We used a search strategy that included the key concepts related to our research question: WE, Korsakoff syndrome, and dysphagia. Consequently, our PubMed search was: ((wernicke encephalopathy[MeSH Terms]) OR (korsakoff syndrome[MeSH Terms])) AND (dysphagia[MeSH Terms]). Searches in Scopus used similar versions of these terms appropriate for this specific database. As we aimed to generate an extensive list of articles suitable for answering our research question, we did not apply any search filters. Moreover, we did not apply any language restrictions to our search.

Two authors reviewed the title, abstract, and full text (when needed) of all retrieved articles and assessed whether the study met the inclusion criteria. A third reviewer’s opinion was considered if disagreements were not solved through discussion.

The PCC mnemonics for this systematic review were: children and adults (over 18 years old) (P), with studies investigating patients with dysphagia (C), in the context of WE (C). We planned to include prospective and retrospective observational and interventional studies.

We excluded conference abstracts, commentaries, and opinions. We also excluded narrative reviews, but we examined their reference lists for possible inclusions. 

We extracted data to a pro forma template initially piloted on a set of five randomly selected articles and adjusted as necessary. One reviewer extracted all relevant information, and a second reviewer checked the data. 

Our main scope was to provide an overview of the evidence that has been reported on dysphagia as a WE symptom, regardless of the risk of bias in the included studies [20]. Therefore, we did not perform a formal evaluation of the methodological quality of the included studies.

### 3.2. Results

**Figure 1:**
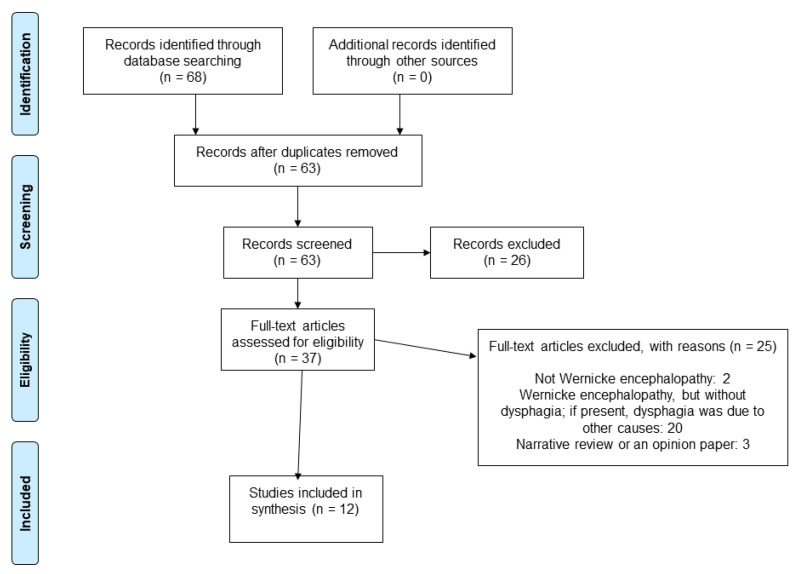
Flow chart showing the process for inclusion of studies.

Our search resulted in 68 records. Five duplicates were removed, so a total of 63 unique studies were assessed in full text; 12 papers were ultimately included [15,24,25,26,27,28,29,30,31,32,33,34]. The PRISMA diagram with the selection process of the studies is presented in Figure 1.

Twenty-five articles were excluded for the following reasons: patient did not have WE (n = 2); patient had WE but without dysphagia; if present, dysphagia was due to other causes (e.g., psychiatric illness, esophageal disease) (n = 20); article was a narrative review or opinion paper (n = 3). 

The characteristics of the included case reports are summarized in Table 1.

This systematic review included 12 cases of patients aged between 12 and 81 years with WE and dysphagia. Most of the patients were males (9/12, 75%). The years of publication ranged from 1997 through 2021.

If we include our patient in this case series, dysphagia was present at the onset of WE in 9/13 individuals (69.23%) [24,25,26,27,28,29,33,34]. Among them, three patients also presented with the classic WE triad of cognitive impairment, ocular abnormalities, and ataxia [27,29,34]. All three of these characteristic clinical signs were present in only 5/13 (38.46%) patients [27,29,30,34]. Ocular abnormalities were present in 9/13 (69.23%) cases [15,24,27,28,29,30,33,34], ataxia was present in 9/13 (69.23%) cases [15,24,27,29,30,32,33,34], and cognitive dysfunction was present in 9/13 (69.23%) cases [15,26,27,29,30,31,32,34]. 

The patients had various underlying pathologies, including alcoholism [33], illicit drug abuse [24], gastrectomy [25,28,30,34], Crohn’s disease [27], parenteral nutrition [26], hyperemesis gravidarum [15], thyroid disease [31], and prolonged fasting [29]. One case had a previous influenza infection [32]. Our case had a history of respiratory infection and prolonged fasting. 

Neuroimaging data were available for all patients except one [33]. The brain MRIs were normal in two cases [24,28]. In addition, the patient described in the present case report had no lesions suggestive of WE. Brain abnormalities were reported in 10/12 patients (83.33%). Most frequently, the lesions were located in the bilateral thalami [15,26,29,30,31,32,34] and periaqueductal gray matter [15,29,30,31,32,34]. Other sites of cerebral lesions included the mamillary bodies [26,31], hypothalamus [15], tectal plate [31,32], floor of the fourth ventricle [29], and vestibular nuclei [30]. 

All patients received thiamine in different doses, with improvement of symptoms (see Table 1).

## 4. Discussion

This systematic review identified 12 cases of WE with dysphagia. We reported our own additional case of thiamine deficiency with dysphagia at the onset. 

In our patient, the onset of dysphagia and abducens nerve palsy following a respiratory infection was particularly challenging to diagnose. The brain MRI revealed left otomastoiditis, which can cause Gradenigo’s syndrome, in the context of the spread of an otic infection into the apical part of the petrous temporal bone (petrous apicitis) [35]. Classically, Gradenigo’s syndrome consists of unilateral facial pain (V nerve involvement), lateral gaze paralysis (VI nerve involvement), and otorrhea [36]. However, various atypical manifestations have been reported, including presentations with IX and X nerve palsies without involvement of the trigeminal nerve [35]. Cerebral CT and MRI did not reveal any temporal bone inflammation in the present case. 

The differential diagnosis also included Miller–Fisher syndrome, a variant of Guillain–Barré syndrome. Miller–Fisher syndrome is associated with upper respiratory tract infections and presents with ophthalmoplegia, ataxia, and areflexia [37]; it is occasionally associated with sensory symptoms in the limbs. The autoimmune process responsible for Miller–Fisher syndrome can affect both the oculomotor and lower cranial nerves [38], with mean recovery times between 8 and 12 weeks [38]. Our patient had the classic triad of Miller–Fisher syndrome, as well as a history of infection. However, antibodies against the GQ1b ganglioside were negative, and nerve conduction studies excluded a Guillain-Barré variant. Moreover, a marked improvement after treatment with thiamine ruled out this diagnosis. 

Another diagnosis that was considered was foodborne botulism, which is caused by *Clostridium botulinum*, a neurotoxic, anaerobic, Gram-positive bacillus. In this disease, neurotoxins are absorbed from the gastrointestinal tract, disseminate hematogenously, and cause an irreversible blockade of the peripheral cholinergic nerve terminals, including the neuromuscular junctions, sympathetic and parasympathetic ganglia, and parasympathetic postganglionic sites [39]. The symptoms of botulism include digestive complaints (e.g., constipation, vomiting, abdominal cramps) and neurologic manifestations, initially with ophthalmologic and bulbar signs, including dysphagia. However, the key distinguishing features of botulism are a history of ingesting home-canned foods, dilated, poorly reactive pupils, ptosis, descending flaccid paralysis with preserved reflexes, and absence of cerebellar and cognitive signs [39].

Central pontine myelinolysis is mainly seen in alcoholic patients, but it has been reported in other contexts, including malnutrition, cancer, renal disease [40], and rapid correction of hyponatremia. The symptoms include oculomotor nerve palsy, dysphagia, dysarthria, weakness in the extremities, altered tendon reflexes, and confusion [40]. This diagnosis was excluded because our patient had normal plasma sodium concentrations throughout the course of her illness.

Our patient presented with dysphagia at the onset, which was the same as 8/12 cases we found in the literature [24,25,26,27,28,29,33,34]. However, only three of those eight individuals developed the typical symptoms of WE (ocular abnormalities, ataxia, and confusion) [27,29,34], as was observed in our patient. The other 4/12 patients developed dysphagia after their other WE symptoms [15,30,31,32], and two of those four had the WE triad [15,30]. 

Signs of peripheral nerve involvement (e.g., decreased tendon reflexes) were reported in 4/12 cases [15,24,30,33]. Hence, among the 12 literature cases, none went through the same pattern of impairment as our patient, who had dysphagia at the onset, followed by ocular abnormalities, ataxia, cognitive impairment, and then dry beriberi. The combinations of clinical signs in the 12 cases were very heterogeneous, suggesting that further research is needed to distinguish a pattern of cerebral involvement in patients with WE and dysphagia and to understand why some patients develop particular symptoms while others do not.

Brain MRI can detect characteristic findings of WE, and some authors consider it more sensitive for detecting WE lesions in non-alcoholic patients than in alcoholic patients [41]. It has a high specificity of 93% but a poor sensitivity of 53% [42]. Classic brain MRI findings in WE patients include bilateral lesions in the mammillary bodies, thalamus, and periaqueductal and periventricular gray matter, and collicular bodies [42]. However, MRI may also reveal uncommon sites of lesions, such as the pre-and postcentral gyri, putamen, caudate, splenium of the corpus callosum, red nucleus, substantia nigra, dorsal medulla, pons, cranial nerve nuclei, vermis, dentate nucleus, and the paravermian region of the cerebellum [43].

Altered mental status is the most common symptom in WE, occurring in 34–82% of cases [44]. It may arise from damage to the reticular system at the level of the midline thalamic nuclei or mammillary bodies [45]. The second most common clinical manifestation in WE is oculomotor impairment, resulting from lesions of the pontine tegmentum, including the abducens and oculomotor nuclei. The third most common symptom, ataxia, is due to involvement of the cerebellar vermis and vestibular dysfunction. Other less frequent manifestations of WE, including dysphagia, are probably due to lesions in different brain regions, such as the brain stem regions. Neuroimaging studies have reported different patterns of brain lesions in alcoholic and non-alcoholic patients [46], with cranial nerve nucleus involvement representing a distinctive pattern in non-alcoholic patients. However, the mechanism of these specific findings remains unclear. Interestingly, in our case series with dysphagia, only 1/13 of the patients had a history of alcoholism [33].

Our patient did not display the classic lesions on MRI, as the typical MRI findings of WE are not observed in all patients. In the case series of this review, the lesions were most frequently located in the thalamus and periaqueductal gray matter. 

Deglutition is controlled by the glossopharyngeal, vagal, and hyoglossal nerves. The motor nuclei of these cranial nerves are located in the floor of the fourth ventricle, a region reportedly affected in WE [47]. However, in our series, only one patient displayed lesions in this area on MRI [29]. 

Considering the pathophysiology of WE symptoms, in our case, the thiamine reserves were depleted due to anorexia and caloric restriction. Without adequate consumption, the body’s thiamine stores become depleted within 3 to 4 weeks. Therefore, the function of the thiamine-dependent enzyme systems deteriorated, and thiamine levels decreased. Brain cells depend on thiamine as a coenzyme for various metabolic processes. Thiamine is essential for the metabolism of carbohydrates to produce cellular energy, lipid metabolism for the integrity of the myelin sheath, and metabolism of amino acids for adequate neurotransmitter synthesis and function. Cellular damage occurs as early as 4 days after thiamine depletion if these metabolic processes are altered [11,48]. Without adequate correction, metabolic dysfunction progresses, leading to cellular death. Cerebral lesions develop in approximately 14 days. 

Various concerns have been raised about the high possibility of bias associated with case reports and the weak inferences they may provide. Our findings may be restricted by the quality and extent of the data provided in the case reports, which may not have been consistent among the 12 included articles. Furthermore, individual case reports are low on the pyramid of evidence because they consider single patients rather than patient groups and therefore have no statistical power. 

Nevertheless, case reports are considered a relevant, pertinent, and requisite study design in promoting scientific research, particularly for rare conditions. Despite the methodological limitations of case studies in analyzing treatments and developing new tests, observing individual patients can provide valuable insights into etiology, pathogenesis, natural history, and treatment [23]. Case studies are an essential basis for learning by pattern recognition [23]. Moreover, they are indispensable for shedding light on new events and providing the first-line evidence needed to test hypotheses with statistical methods. 

For example, in the 1960s, there was an epidemic of babies with severe birth defects of unknown origin. Australian doctor McBride published a case series of babies with birth defects, which indicated that thalidomide, taken by their mothers to fight nausea, may have induced the birth defects [49]. His hypothesis led to interruption of the drug being given to pregnant women and was later demonstrated to be correct.

There is an increasing trend to include case reports/series in systematic reviews and to value their role in research. For instance, a systematic review of lipodystrophy cases enabled the core and supportive clinical features of the condition to be proposed and the data on available treatments to be presented [50]. Another systematic review of cases of glycogenic hepatopathy, a rare disorder, warranted the characterization of patterns of liver enzyme abnormalities and hepatic injury [51]. Furthermore, compared to cohort studies, case reports include much more data on individual patients [52]. 

Our case highlights the necessity of having a high index of suspicion of WE, especially in non-alcoholic patients, as the clinical manifestations can be variable and mimic several other pathologies. In addition, although rarely reported in WE, dysphagia may be notably problematic, as it could lead to further inadequate oral intake and further aggravation of the disease. Furthermore, dysphagia can be an important symptom in digestive diseases; therefore, it may be overlooked and considered a symptom of the causative pathology for WE.

To the best of our knowledge, this is the first systematic review of dysphagia as a symptom of WE. We found that, to date, only 12 cases of dysphagia as a symptom of WE had been reported, with swallowing problems being present at the onset in only nine patients (including the current case report).

## 5. Conclusions

Our systematic review found that dysphagia is a rare symptom of WE, suggesting that thiamine deficiency should be suspected in patients with dysphagia of unknown cause, even in the absence of alcohol abuse. In contrast to most WE patients, the majority of patients included in this review presented with dysphagia at the onset of their illness, even in the absence of the classic triad of cognitive impairment, oculomotor abnormalities, and ataxia, indicating that there could be varying susceptibility for clinical manifestations of thiamine deficiency in different brain regions.

## Figures and Tables

**Table 1:** Characteristics of the included studies.

Study	Age (Years)	Gender	Neurological Symptoms and Signs	Laboratory Tests	Neuroimaging	Treatment and Outcome	Notes
Antel 2015 [24]	22	Male	Bilateral ophthalmoplegia and ptosis, dysphagia, severe spastic anarthria without any volitional movement of the bulbar musculature. Ataxic gait. Absent tendon reflexes. Able to communicate in writing. Respiratory insufficiency.	All laboratory investigations were normal, including thyroid tests, liver function tests, and CSF examination. HIV and syphilis serology were negative.	Normal brain MRI.	IV immunoglobulin and prednisone without clinical improvement. IV thiamine (100 mg 8-hourly) for 7 days, then 100 mg daily orally. The patient responded within days, with resolution of eye signs and recovery of bulbar function. PEG was removed within 1 month.	Dysphagia at onset. Illicit substance abuse. Non-alcoholic. Severe vomiting and diarrhea the week before admission.
Arita 2015 [25]	58	Male	Dysphagia.	N/A	MRI: suggestive of Wernicke encephalopathy.	IV thiamine After thiamine treatment, dysphagia improved.	Dysphagia at onset. Non-alcoholic. Distal gastrectomy for gastric cancer. Vomiting, anorexia.
Cefalo 2013 [26]	12	Male	Acute and rapidly progressive alteration of mental status consisting of hallucinations, aphasia, dysphagia, tremors, bilateral mydriasis, and impairment of consciousness.	Low thiamine level (31.6 nM/L).	MRI: bilateral and symmetric high T2 signal in the thalamic region and mammillary bodies.	IV thiamine (500 mg for 5 days), followed by intramuscular injection (100 mg/day for 20 days and then 100 mg three times a week), with slight neurologic improvement. Oral lorazepam (0.08 mg/kg) with abrupt change in vigilance, with full consciousness, recovery of spontaneous speech, progressive capability of oral alimentation, increase of movements, and postural changes.	Dysphagia at onset. Primitive fronto-parietal neuroectodermal tumor with resection. Chemotherapy. Radiotherapy. Autologous peripheral hemopoietic stem cell rescue therapy. Prolonged parenteral nutrition.
Delavar Kasmaei 2014 [27]	41	Male	Diplopia, dysarthria, dysphagia, followed by gait disturbances and progressive ataxia accompanied by confusion, apathy, and disorientation. Bilateral horizontal nystagmus in lateral gaze, left abducens nerve palsy, upward gaze palsy. Absent gag reflex.	N/A	MRI: changes consistent with Wernicke encephalopathy.	Thiamine led to partial resolution of his upward gaze palsy and nystagmus on the first day. At the end of the third day of treatment, except for gate ataxia, all other symptoms were fully corrected, and he was totally conscious. After the fifth day, his gait became normal, and after one week, he was discharged in good general condition.	Dysphagia at onset. Non-alcoholic. Severe nausea and vomiting. Untreated Crohn’s disease.
Dirani 2017 [28]	20	Male	Dysphagia. Diplopia. Bilateral 6th nerve palsy, nystagmus. Bifrontal headache, photophobia, and phonophobia with mildly reduced visual acuity.	Low levels of vitamin D and vitamin B1 and microcytic anemia.	MRI: normal.	IV thiamine (500 mg every 8 h for 2 days) and 500 mg intramuscularly once daily for an additional 5 days in combination with magnesium and other vitamins in the B group. Symptoms improved gradually.	Dysphagia at onset. Non-alcoholic. Laparoscopic sleeve gastrectomy. Persistent vomiting 3 weeks after surgery. Fistula with thoraco-abdominal abscess (5 weeks after surgery).
Karaiskos 2008 [29]	44	Male	Bilateral abducens nerve palsies with coarse horizontal and vertical upbeat nystagmus. Dysphagia, dysarthria. Dense amnestic deficit consistent with Korsakoff syndrome. Ataxia.	CSF: normal.	MRI: symmetric high signal intensities in the medial thalami, periaqueductal gray matter, and in the floor of the 4th ventricle on T2 and FLAIR sequences.	IV thiamine (100 mg/day) IV antibiotics. Improvement. The gastrostomy tube was removed 8 days after initiation of thiamine.	Dysphagia at onset. Non-alcoholic. Severe malnutrition, prolonged fasting. Bilateral pleural effusions and a pericardial effusion. Fever. Slow onset and progression.
Kikuchi 2000 [30]	68	Male	Numbness in the feet, followed by dysphagia, unsteady gait, and diplopia. Total ophthalmoplegia. Absence of doll’s eye movement. Absent deep tendon reflexes. Short-term memory impairment.	Serum thiamine level: 9 ng/mL (normal range 20–50 ng/mL).	Symmetrical high intensity lesions in the periaqueductal area of the midbrain, dorsomedial nuclei of bilateral thalami, and vestibular nuclei.	IV thiamine. Marked improvement.	Non-alcoholic. Proximal subtotal gastrectomy and reconstructive surgery of the jejunal interposition for gastric cancer. Fever. Slow onset, chronic progression, and then rapid worsening after fever.
Mutti 2021 [31]	65	Male	Apathy, forgetfulness, abulia, and mild cognitive impairment, leading to serious malnutrition. Dysphagia.	Hypoalbuminemia. Mild iron deficiency anemia. Suppressed TSH level, high levels of thyroid hormones (FT3 = 12.90 pg/mL, FT4 = 5.09 ng/dL), high positive values of anti-thyroid receptor antibodies (antiTSH-R = 35.55 uU/L). Hypotension, sinusal tachycardia.	MRI: bilateral and symmetrical FLAIR and T2-hyperintense lesions diffused along the periaqueductal area, tectal plate, thalami, and mamillary bodies.	Parenteral nutrition enriched with multivitamins and minerals (in the following months). Twenty-four months after discharge, he recovered from his psychiatric and focal neurological symptoms, except ataxia.	Non-alcoholic. Solitary thyroid nodule and thyroiditis. Central pontine myelinolysis (1 month later).
Ros Forteza 2019 [32]	81	Female	Gait impairment. Hypotonia. Somnolence, disorientation to time but not space, incoherent speech. Strabismus (exotropia of the right eye), isochoric and reactive pupils (preserved photomotor and consensual reflexes), persistent horizontal-rotary nystagmus, dysphagia for liquids.	CSF: normal. Serum thiamine level: 27 ng/mL (normal range 28–85 ng/mL). Anemia. Vitamin B12: 158 pg/mL (187–883 pg/mL); vitamin D: 17 ng/mL (30–100 ng/mL); magnesium: 1.37 mg/dL (1.6–2.6 mg/dL); sodium: 135 mg/dL (136–145 mg/dL); proteins: 5.3 g/dL (6.4–8.3 g/dL), and albumin: 2.9 g/dL (3.2–4.6 g/dL).	Lesion in the periaqueductal gray matter and tectum and bilateral thalamic lesions.	Thiamine 500 mg IV every 8 h (2 days), 500 mg IV every 24 h (5 days), then 100 mg IV every 8 h during the remaining hospitalization period. Multivitamin solution (vitamins A, B, H (biotin), and F). Protein-calorie supplementation. A significant improvement was noted at 3 months.	Non-alcoholic. Hiatal hernia diagnosed 18 years previously, anti-reflux surgery 15 years previously, cholecystectomy, and acute biliary pancreatitis. Three weeks after influenza infection, anorexia, dehydration, mental confusion, altered sleep–wake cycle, and visual and gait impairment.
Sakakibara 1997 [15]	24	Female	Staggering gait, ataxia. Vertigo, ophthalmoplegia, ptosis, diplopia, facial paresis, gaze-evoked nystagmus, dysphagia, dysarthria, weak neck flexion. Diminished tendon reflexes. Memory disturbance. Mild dyspnea. Urge urinary incontinence that changed to reflex-type incontinence.	CSF: normal. Nerve conduction studies: decreased motor action potential in the deep peroneal nerve and absent F wave in the median, tibial, and deep peroneal nerves, suggesting mild polyneuropathy.	Abnormal intensities in medial thalamic/ hypothalamic regions and periaqueductal area.	Double filtration plasmapheresis (Guillain–Barre’ syndrome). Thiamine 100 mg/day (6 weeks). Six weeks after the administration of thiamine, incontinence and neurological signs disappeared almost completely.	Non-alcoholic. Pregnant with hyperemesis gravidarum.
Truedsson 2002 [33]	62	Male	Gait ataxia. Lateral rectus palsy, horizontal nystagmus, dysphagia. Absence of deep tendon reflexes, positive Babinski’s sign on the right side.	Plasma ASAT: 2.65 μkat/L (normal level < 0.80 μkat/L), ALAT: 2.02 μkat/L (normal limit < 0.80 μkat/L), amylase: 0.88 μkat/L (normal range 0.20–0.80 μkat/L), erythrocyte sedimentation rate: 75 mm/h (normal level < 22 mm/h), serum α1-antitrypsin: 1.93 g/L (normal range 0.97–1.68 g/L), serum orosomucoid: 1.61 g/L (normal range 0.54–1.17 g/L). Serum C-reactive protein: 8.9 mg/L (normal limit: < 3.0 mg/L), serum ceruloplasmin: 0.53 g/L (normal range 0.22–0.38 g/L), serum immunoglobulin IgA: 6.30 g/L (normal range 0.70–3.65 g/L), plasma creatinine: 106 μM (normal range 63–105 μM), plasma sodium: 134 mM (normal range 136–146 mM).	N/A	IV glucose. Two injections of thiamine (50 mg/mL, 100 mg per dose) IV. The period between the two doses was 16 h. Neurological signs disappeared after 24 h.	Dysphagia at onset. Alcoholism. A 10 kg weight loss. Emphysema. Fatty liver.
Truong 2016 [34]	27	Female	Confusion, dysphagia, ataxia, dizziness, diplopia and blurred vision. In 3 days, she developed tetraparesis, facial diparesis, horizontal nystagmus, and vigilance troubles.	Serum thiamine: 15.9 ng/mL (normal range 20–100 ng/mL). CSF: increased proteins (509 mg/L; normal range 150–450 mg/L). Electroneuromyography: normal in four limbs.	MRI: high-intensity Flair in the periaqueductal region and bilateral paraventricular regions of the thalami.	IV thiamine (750 mg/day for 2 weeks, followed by 500 mg/day oral). After 1 month, the patient recovered normal vigilance. Diplopia and visual abnormalities, facial diparesis, and dysphagia disappeared. After a follow-up of 14 months, she still had ataxia and cognitive deficits.	Dysphagia at onset. Non-alcoholic. Sleeve gastrectomy (2 months earlier). One month after surgery, she presented with nausea and recurrent vomiting.

ASAT: aspartate aminotransferase; ALAT: alanine aminotransferase; PEG: percutaneous endoscopic gastrostomy.

## Data Availability

The de-identified data of the case report are available on request from the corresponding author. All data for the systematic review are available within the article.

## Supplementary Materials

The following supporting information can be downloaded at https://www.mdpi.com/article/10.3390/nu14245294/s1: Table S1: Laboratory serum tests.
