# Supplementary material for: Wernicke Encephalopathy Presenting with Dysphagia: A Case Report and Systematic Literature Review
Source: Nutrients. 2022 Dec 13;14(24):5294. doi: 10.3390/nu14245294 (PMC9788281; doi:10.3390/nu14245294)
Supplement: Supplementary file 1 [file nutrients-14-05294-s001.zip › nutrients-2084581-supplementary.pdf]

Supplementary Material Table S1. Laboratory serum tests.

**Laboratory test**	**Result**	**Normal range**
Leukocytes	10.800	4.0-9.5 /µL
Lymphocytes	34.3%	20-40%
Granulocytes	63.5%	45-70%
Monocytes	2.2%	3.5-9.5%
Erythrocytes	4.58	4-5.5 x10^6/µL
Hemoglobin	27.5	27-32 pg
Thrombocytes	379	379 x 10^3/µL
Creatinine	0.7	0.7-1.2 mg/dl
Urea	13	15-36 mg/dl
Uric acid	2.2	2.5-6.2 mg/dl
Sodium	140	135-145 mmol/l
Potassium	5.4	3.5-5.1 mmol/l
ALT	9	0-35 U/L
AST	18	14-36 U/L
GGT	21	12-43 U/L
Triglycerides	73	0-200 mg/dl
Cholesterol (total)	227	150-200 mg/dl
LDL cholesterol	148	0-150 mg/dl
HDL cholesterol	65	40-150 mg/dl
Bilirubin	0.4	0.2-1.3 mg/dl
Glycemia	111	74-106 mg/dl
Proteins	6.7	6.4-8.2 g/dl
Albumin	3.0	3.4-5.0 g/dl
CK	47	30-135 U/L
CK-MB	9	0-16 U/L
LDH	145	120-246 U/L
Erythrocyte sedimentation rate	45	2-20 mm/1 h
C reactive protein	28	0-10 mg/l
Fibrinogen	478	200-393 mg/dl
Rheumatoid factor	9.38	0-15.9 U/ml
Interleukin 6	3.75	0-6.65 pg/ml
Procalcitonin	0.036	<0.5 ng/mL
TSH	0.165	0.55-4.78 mU/L
FT3	5.1	3.54-6.47 pmol/l
FT4	15.75	11.5-22.7 pmol/l
Antithyroglobulin antibodies	<1.3	0-4.5 U/ml
Thyroid peroxidase antibodies	28	0-60 U/ml
Vitamin D	25.17	30-100 ng/ml
HIV test	Negative	Negative
Ferritin	103	4.5-170
Anti-streptolysin O antibodies	81	0-408 U/ml
Venereal disease research laboratory (VDRL) test	Negative	Negative
